# Supplementary material for: Reassessing the observational evidence for nitrogen deposition impacts in acid grassland: spatial Bayesian linear models indicate small and ambiguous effects on species richness
Source: PeerJ. 2020 Apr 29;8:e9070. doi: 10.7717/peerj.9070 (PMC7195837; doi:10.7717/peerj.9070)
Supplement: Supplemental Information 1 — Six figures mapping the means and standard deviations of the spatial random fields estimated for all the models fitted in the paper. The final figure illustrates the constrained refined Delaunay triangulation over which the spatial fields are estimated. [file peerj-08-9070-s001.docx]

## Supplementary Information

### SI1: Model spatial random fields

The random fields below plot the spatial autocorrelation that is estimated to remain after accounting for the covariates and monad random effect. Therefore the estimates are dependent on the locations of the sites and plots that are in the model and on the fixed and random independent variables in each model. Note the different scales across the maps of both the means and standard deviations. White crosses indicate site locations.
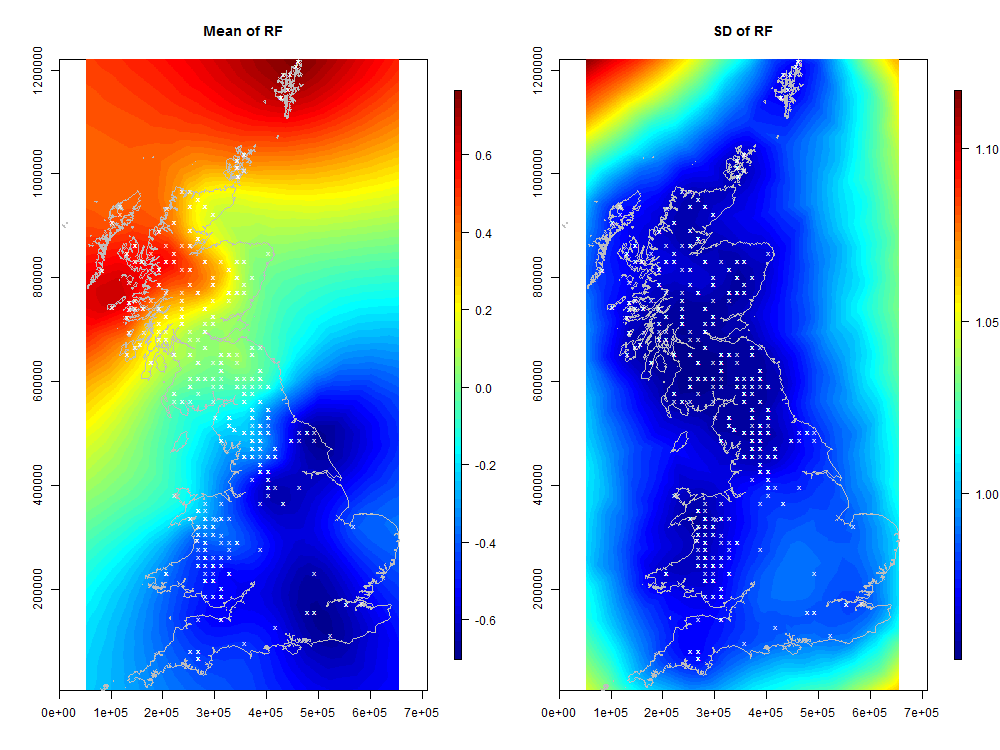


Figure A1.1. MEA10 model (vascular plant richness only). Mean and standard deviation of spatial random fields.


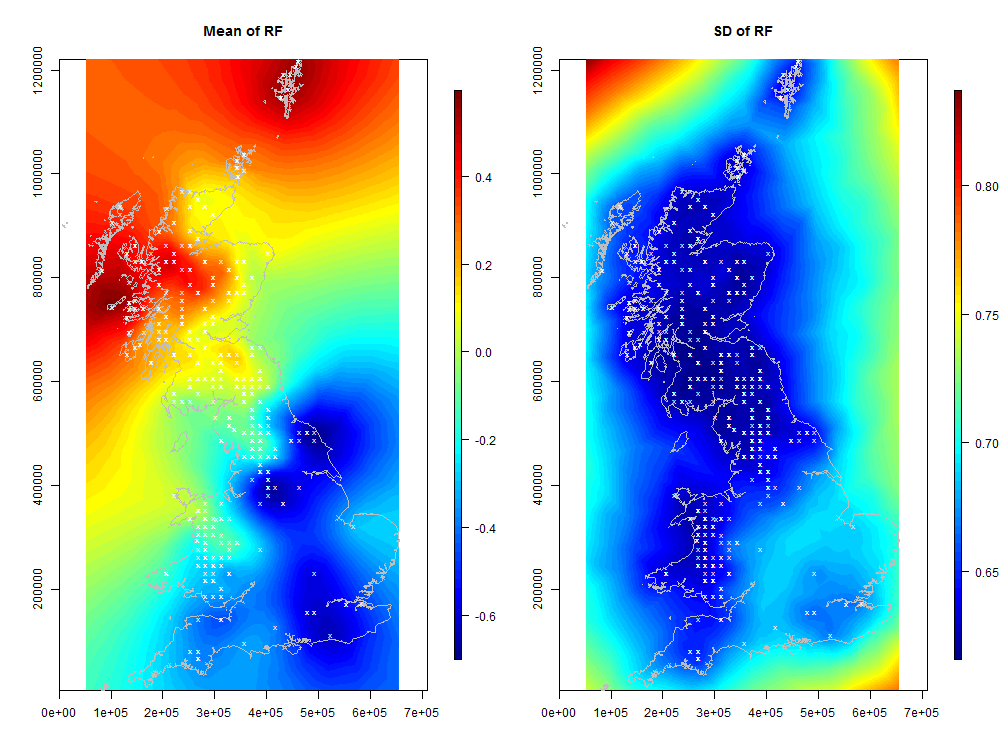


Figure A1.2. MEA10 model (vascular plant and bryophyte richness combined). Mean and standard deviation of spatial random fields.


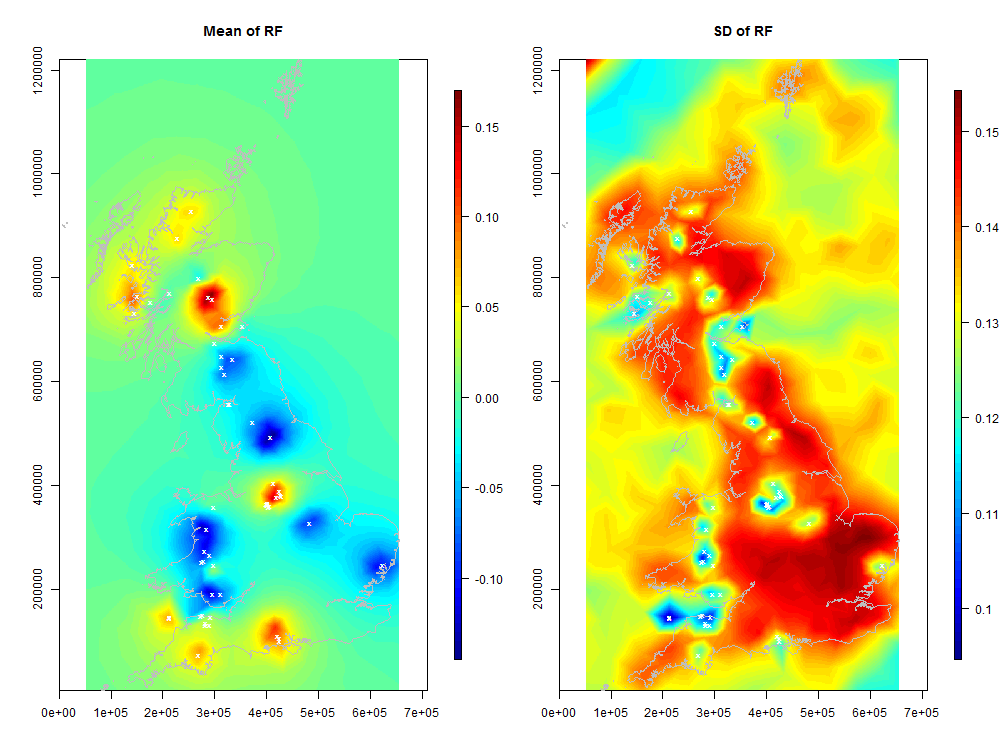


Figure A1.3. SEA04 model 1 (vascular plant richness only). Mean and standard deviation of spatial random fields.


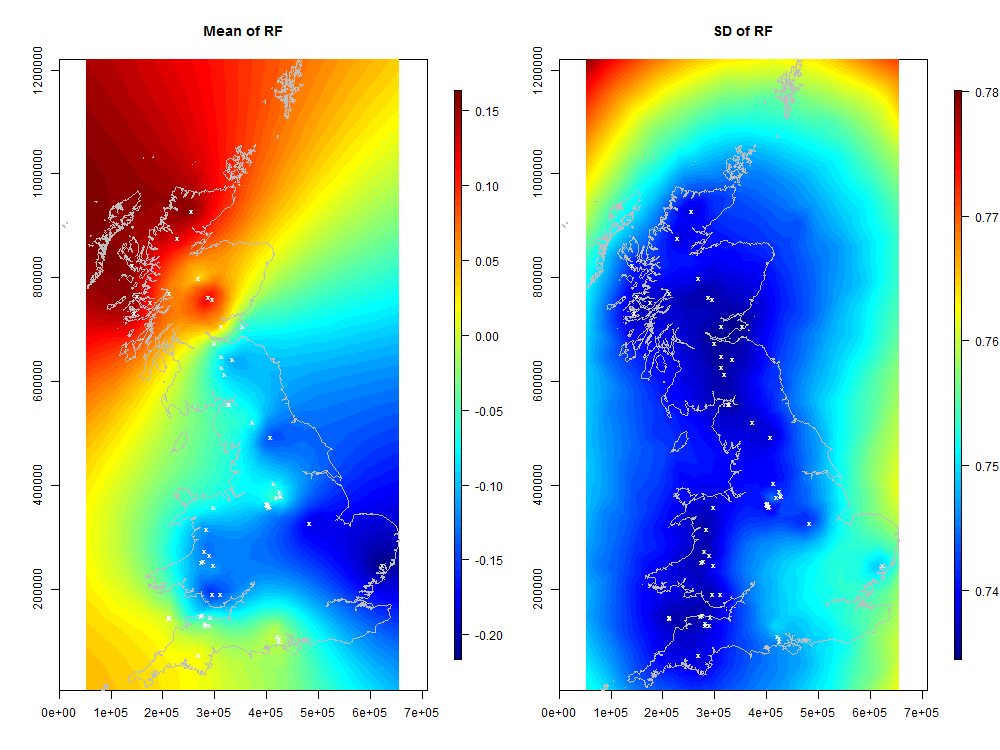


Figure A1.4. SEA04 model 1 (vascular plant and bryophyte richness combined). Mean and standard deviation of spatial random fields.


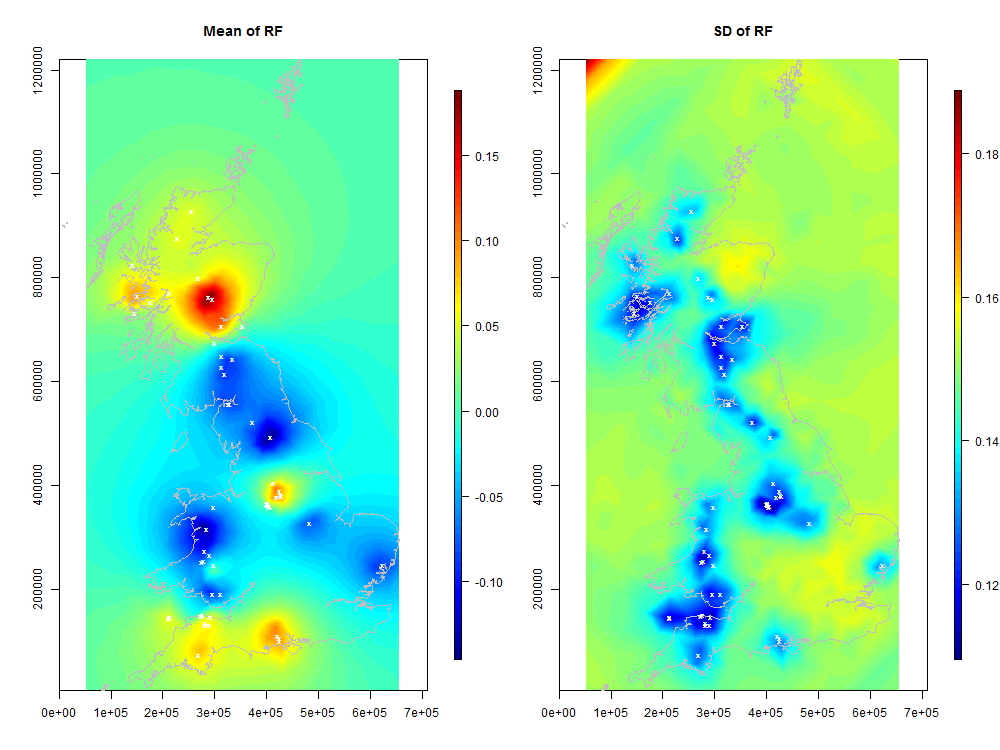


Figure A1.5. SEA04 model 2 (vascular plant richness only). Mean and standard deviation of spatial random fields.


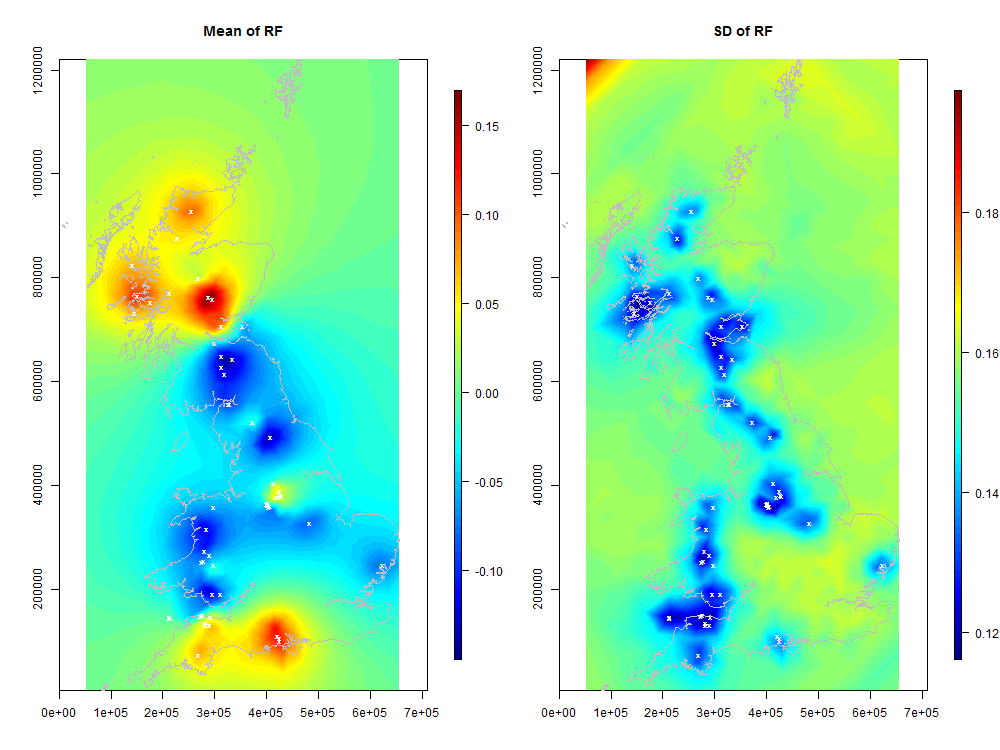


Figure A1.6. SEA04 model 2 (vascular plant and bryophyte richness combined). Mean and standard deviation of spatial random fields.


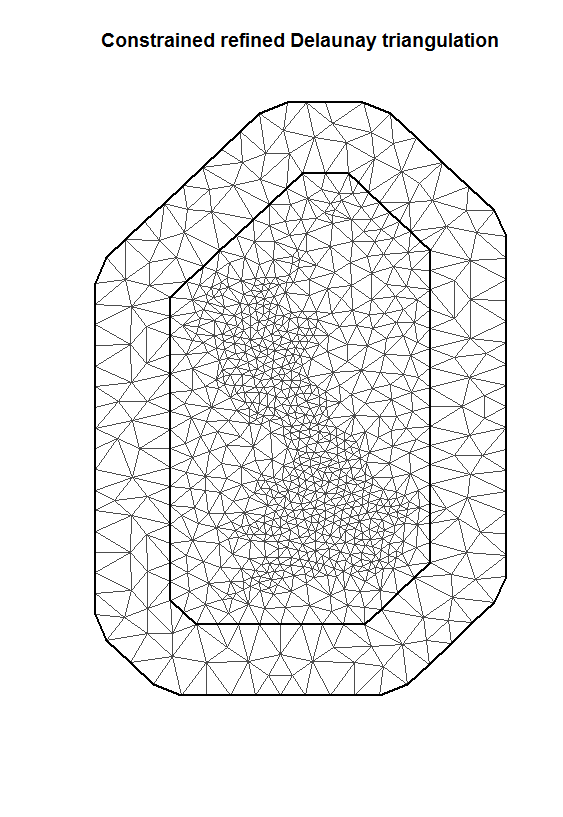


Figure A1.7. Constrained refined Delaunay triangulation of Britain over which the SPDE solution to the zero mean Matern Gaussian Markov random field (GMRF) is estimated.
