# Supplementary material for: Reassessing the observational evidence for nitrogen deposition impacts in acid grassland: spatial Bayesian linear models indicate small and ambiguous effects on species richness
Source: PeerJ. 2020 Apr 29;8:e9070. doi: 10.7717/peerj.9070 (PMC7195837; doi:10.7717/peerj.9070)
Supplement: Supplemental Information 2 — A set of plots providing estimates of regression coefficients for all models fitted in the paper, but where the dependent variable is vascular plant plus bryophyte species richness. [file peerj-08-9070-s002.docx]

### SI2: Regression coefficients from analyses including bryophytes


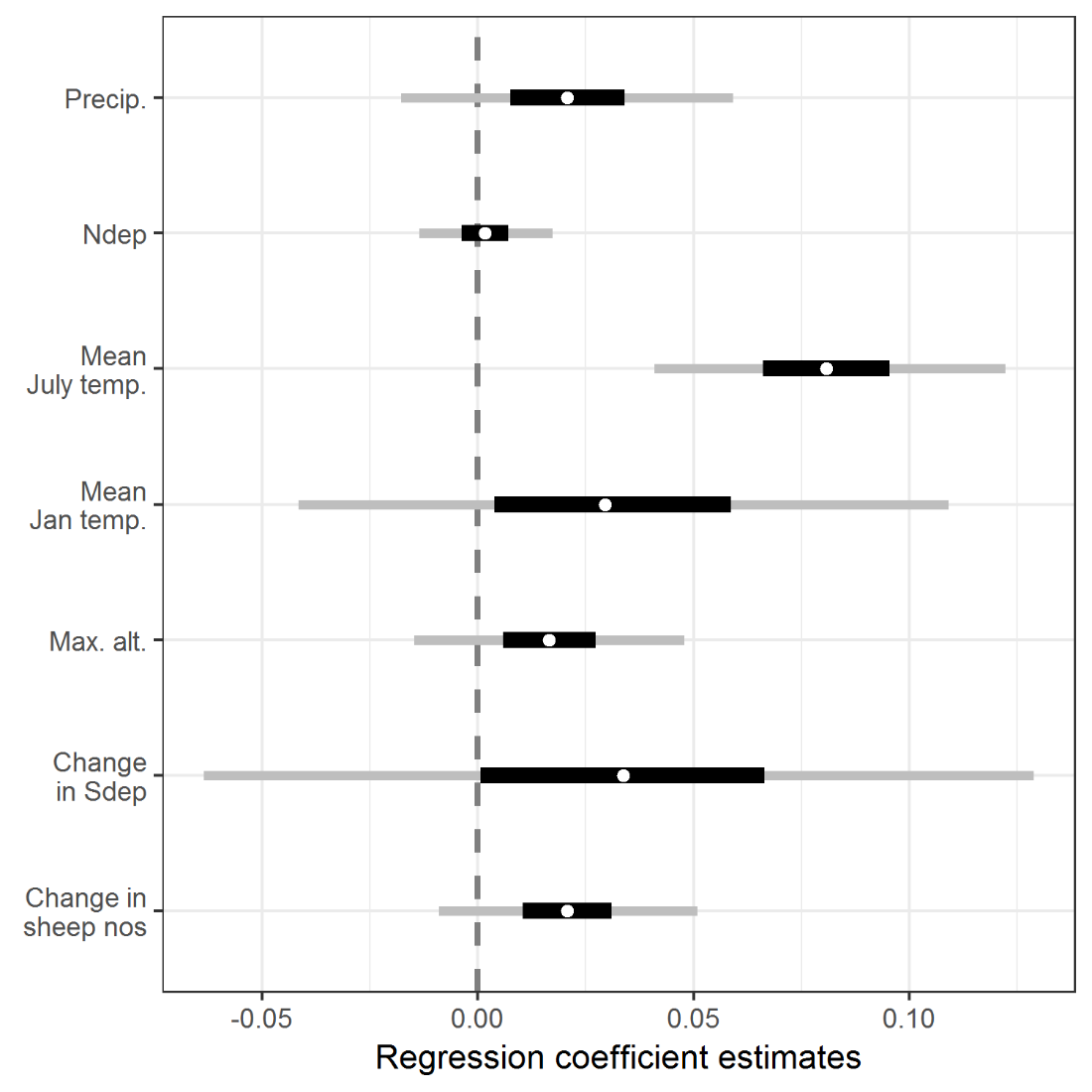


Figure A2.1. Estimated regression coefficients for the reanalysis of Maskell et al. (2010). The dependent variable was vascular plant plus bryophyte species richness. White circles represent the posterior median estimate, black bars the posterior 50% credible interval, grey bars the posterior 95% credible interval. All covariates are described in Table 1.


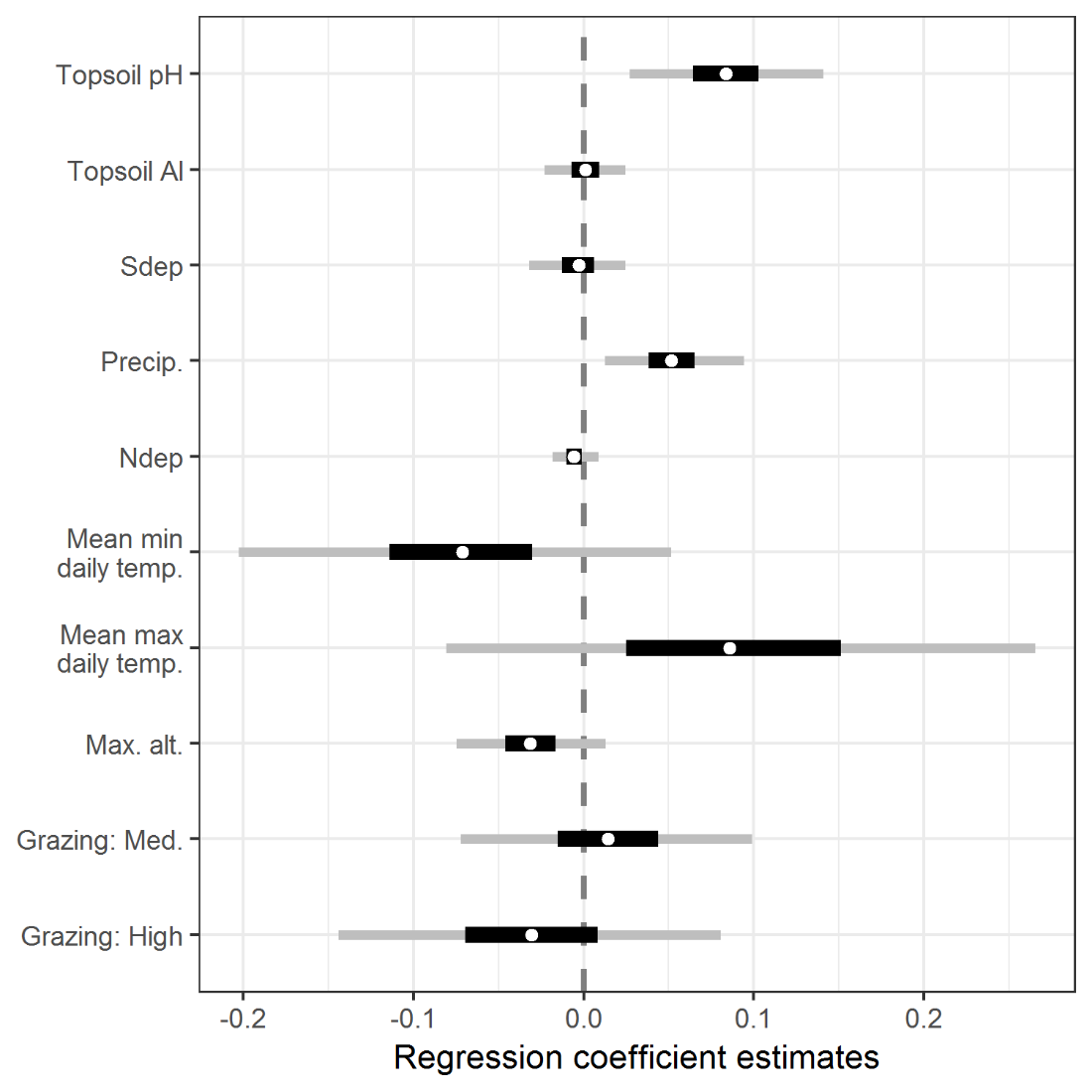


Figure A2.2. Estimated regression coefficients for the reanalysis of Stevens et al. (2004) using a reduced set of covariates chosen for their similar ecological status to the covariates used by Maskell et al. (2010), referred to in this paper as SEA04 model 1. The dependent variable was vascular plant plus bryophyte species richness. White circles represent the posterior median estimate, black bars the posterior 50% credible interval, grey bars the posterior 95% credible interval. All covariates are described in Table 1.


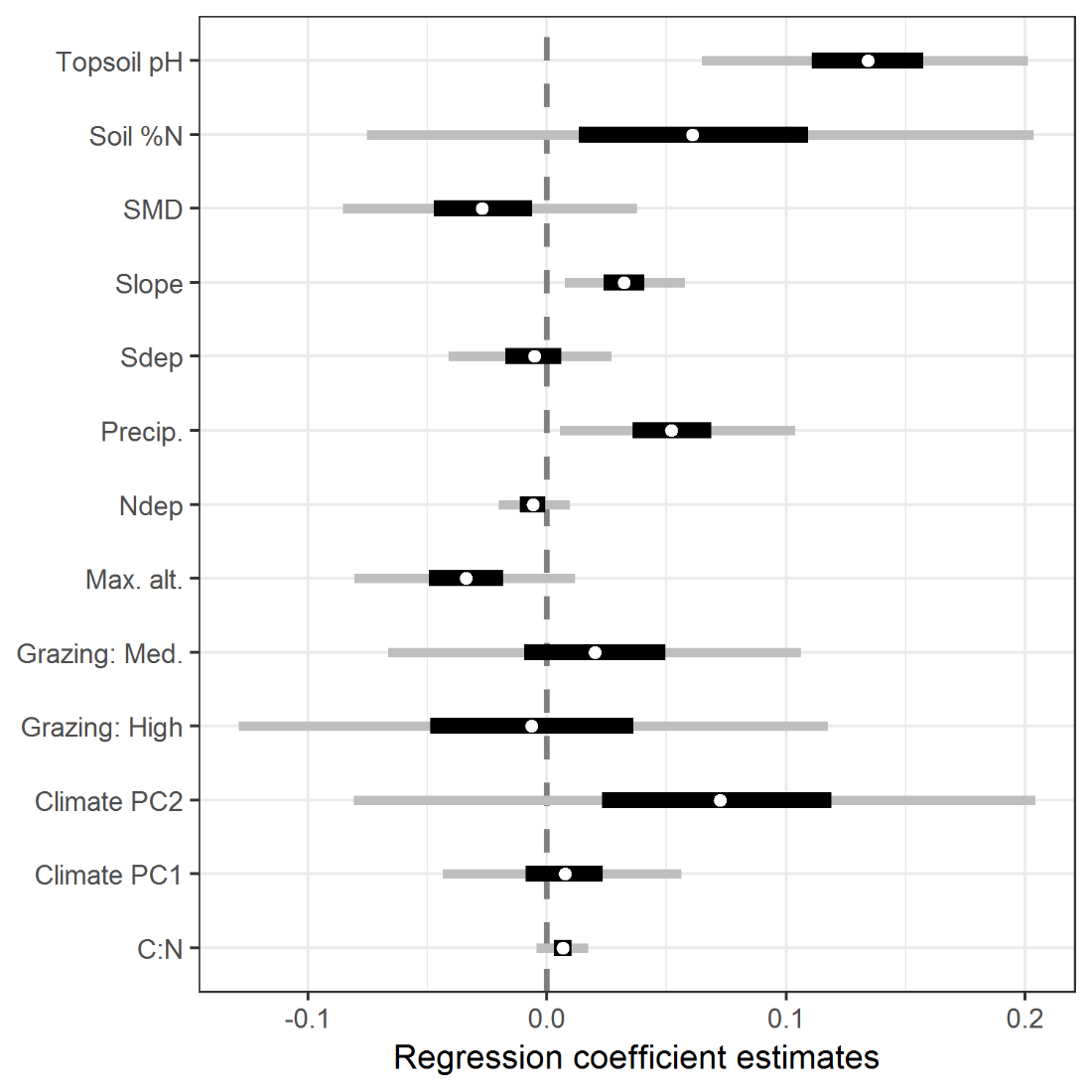


Figure A2.3. Estimated regression coefficients for the reanalysis of Stevens et al. (2004) using a set of covariates designed to match the original analysis of that paper as closely as possible, referred to in this paper as SEA04 model 2. The dependent variable was vascular plant species plus bryophyte richness. White circles represent the posterior median estimate, black bars the posterior 50% credible interval, grey bars the posterior 95% credible interval. All covariates are described in Table 1.
