# Supplementary material for: Reassessing the observational evidence for nitrogen deposition impacts in acid grassland: spatial Bayesian linear models indicate small and ambiguous effects on species richness
Source: PeerJ. 2020 Apr 29;8:e9070. doi: 10.7717/peerj.9070 (PMC7195837; doi:10.7717/peerj.9070)
Supplement: Supplemental Information 3 — Plots of regression coefficients for the post hoc models fitted for the dataset of Maskell et al. (2010). [file peerj-08-9070-s003.docx]

### SI3: *Post hoc* models of MEA10 with partial accounting for spatial autocorrelation

Both models presented here use vascular plant richness only as the dependent variable, and were not the DIC-favoured models for these datasets.

#### 1 km square-level random effect only


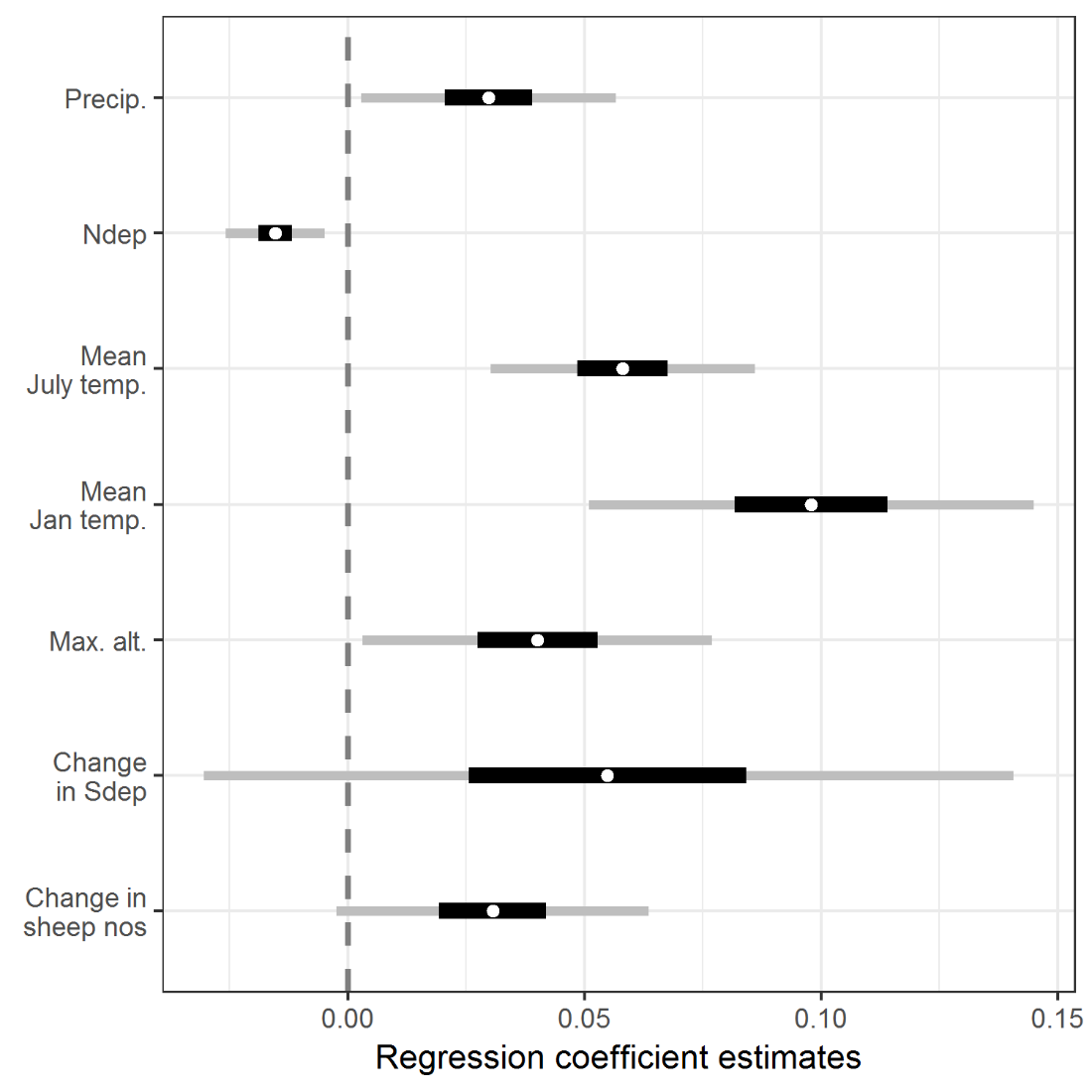


Figure A3.1. Estimated regression coefficients for the reanalysis of Maskell et al. (2010) using a 1 km square-level random effect only. The dependent variable was vascular plant species richness. White circles represent the posterior median estimate, black bars the posterior 50% credible interval, grey bars the posterior 95% credible interval. All covariates are described in Table 1.

#### Spatial mesh only


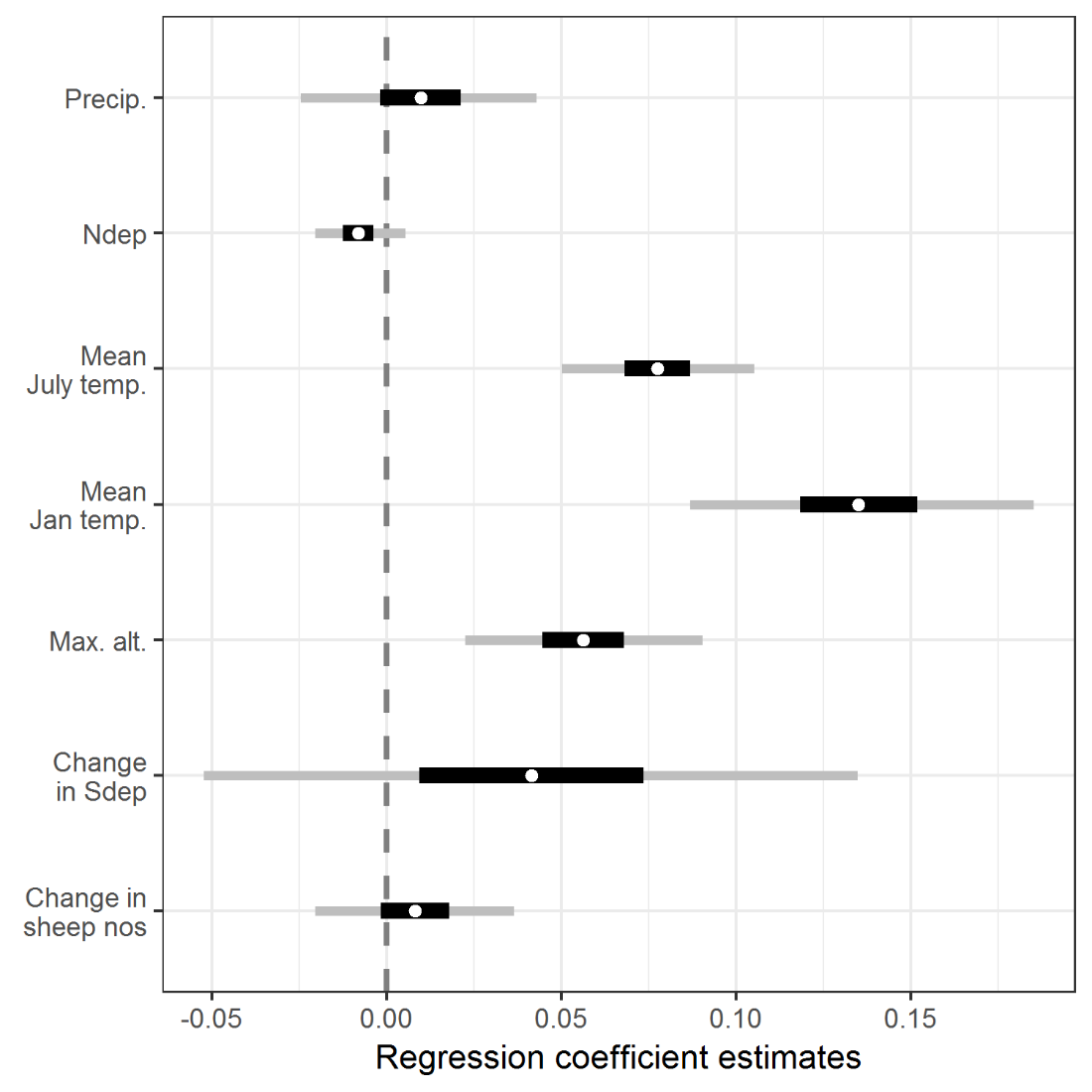


Figure A3.2. Estimated regression coefficients for the reanalysis of Maskell et al. (2010) using the spatial mesh only. The dependent variable was vascular plant species richness. White circles represent the posterior median estimate, black bars the posterior 50% credible interval, grey bars the posterior 95% credible interval. All covariates are described in Table 1.
